# One-Stage Full-Thickness Eyelid Reconstruction Using Nasal Septal Chondromucosal Grafts, Large Local Flaps, and Buccal Mucosal Graft for Donor-Site Repair

**DOI:** 10.3390/jcm15093190

**Published:** 2026-04-22

**Authors:** Ki Hyun Kim, Jeong Hun Ahn, Kyung Min Kim, Sang Seok Woo, Jun Won Lee, Seong Hwan Kim, Jai Koo Choi, In Suck Suh

**Affiliations:** 1-8 Department of Plastic and Reconstructive Surgery, Kangnam Sacred Heart Hospital, College of Medicine, Hallym University, Seoul 07441, Republic of Korea; rlgus0608@hanmail.net (K.H.K.); jeonghun@hallym.or.kr (J.H.A.); baoro333@gmail.com (K.M.K.); ssw1218@gmail.com (S.S.W.); ljw2023@hallym.or.kr (J.W.L.); kalosmanus@naver.com (S.H.K.); jkchoi1957@hallym.or.kr (J.K.C.)

**Keywords:** eyelids, plastic surgery procedures, nasal septum, nasal cartilages, mucous membrane, surgical flaps, transplantation, autologous

## Abstract

**Background**: Eyelid reconstruction is particularly challenging because of the delicate anatomy and its critical functional and aesthetic roles. Although various methods have been described for anterior and posterior lamellar repairs, no standardized approach has been established. We developed a single-stage technique integrating reconstruction of both lamellae. **Methods**: This retrospective case series included seven consecutive patients who underwent full-thickness eyelid reconstruction between 2012 and 2024. Patients were included if they had full-thickness defects requiring reconstruction of both lamellae, underwent reconstruction using a nasal septal chondromucosal graft combined with a large local flap, and had at least 12 months of follow-up. The posterior lamella was reconstructed using nasal septal chondromucosal grafts, and the anterior lamella using large local flaps. Donor sites were managed using various methods. **Results**: All patients (7/7) achieved complete graft survival without partial or total graft loss. All patients achieved complete eyelid closure without lagophthalmos, and no cases of ectropion, corneal complications, or graft failure were observed. Buccal mucosal grafting demonstrated the most favorable donor-site outcomes, with uneventful healing and no septal perforation or airway-related complications. **Conclusions**: This single-stage approach combining chondromucosal grafts and local flaps is a feasible and reproducible option for selected patients, providing reliable structural support and satisfactory functional outcomes.

## 1. Introduction

The eyelid has a complex anatomy and can be divided into anterior and posterior lamellae. The anterior lamella comprises the skin and orbicularis muscles, while the posterior lamella comprises the tarsus and conjunctiva [1].

Reconstruction of eyelid defects caused by tumors, trauma, burns, and congenital factors remains a significant challenge in plastic and reconstructive surgery because of the anatomical complexity, functional considerations, and aesthetic concerns [2,3]. Small partial-thickness defects may be closed primarily. However, larger full-thickness defects, especially those involving more than one-third to one-half of the horizontal lid, require complex reconstructions to restore both the anterior and posterior lamellae [4].

The posterior layer, particularly the tarsal plate, is key to restoring the structural integrity and intact function of the defective eyelid [5]. Various autologous and allogeneic posterior lamellar substitutes have their advantages and disadvantages; however, new materials and tissue engineering are still in the early stages of research. Free composite grafts of the tarsoconjunctiva, hard palate, and nasal chondromucosa have been used for posterior lamellar reconstruction [6].

Unlike skin grafts, composite grafts are much thicker, and only small composite grafts can be sustained purely by the imbibition of nutrients from the wound bed [7]. Haas and Glogau suggested that, for full-thickness defects, composite grafts larger than 1.5 cm have failure rates of >50% [8]. Therefore, no standard for posterior lamellar reconstruction has been established.

Several surgical techniques are available to restore the anterior lamellae of the upper and lower eyelids [9]. For anterior lamella restoration, the Mustarde lid-switch flap with cheek rotation flap, Cutler–Beard lower lid bridge flap, and Fricke flap are the commonly used local flaps [2]. Although various methods for posterior and anterior lamellar reconstruction have been documented individually, reports describing their combined application in a single procedure are scarce [3].

However, no standardized approach has been established for full-thickness eyelid defects requiring simultaneous reconstruction of both lamellae. Furthermore, reports describing integrated single-stage reconstruction of both anterior and posterior lamellae remain limited.

The aim of this study was to evaluate the feasibility and clinical outcomes of a one-stage reconstructive approach using a nasal septal chondromucosal graft combined with large local flaps for full-thickness eyelid defects.

## 2. Materials and Methods

This study was conducted in accordance with the principles of the Declaration of Helsinki and approved by the Institutional Review Board (IRB No. 2025-12-029). Written informed consent was obtained from all patients. All clinical photographs were anonymized to prevent patient identification.

### 2.1. Study Design

This study was designed as a retrospective case series conducted at a single institution. The study period was from March 2012 to December 2024. Following institutional ethical approval, we retrospectively reviewed seven patients who underwent reconstruction for eyelid defects following tumor excision or trauma at our institution between March 2012 and December 2024.

### 2.2. Patients

Data on patient demographics and clinical variables, including age, sex, comorbidities, history of ocular or nasal surgery, defect etiology, defect site, defect size, reconstruction method, and follow-up duration, were collected. All procedures were performed at a single institution by the same surgical team to ensure consistency of the technique.

All consecutive patients who met the inclusion criteria during the study period were included.

Inclusion criteria were as follows:

(1) full-thickness eyelid defects requiring reconstruction of both lamellae
(2) reconstruction using a nasal septal chondromucosal graft combined with a large local flap
(3) at least 12 months of follow-up

Exclusion criteria included primary closure for relatively small defects, staged reconstruction, and insufficient follow-up.

### 2.3. Surgical Technique

**Figure 1:**
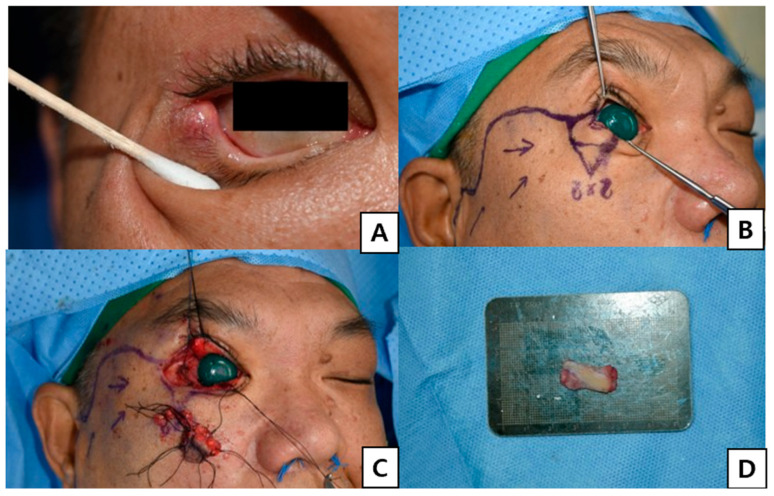
Preoperative findings and posterior lamellar graft preparation. (**A**) Preoperative image. (**B**) Preoperative design. (**C**) Surgical defect after tumor resection. (**D**) Harvested nasal septal chondromucosal graft prepared for posterior lamellar reconstruction.

**Figure 2:**
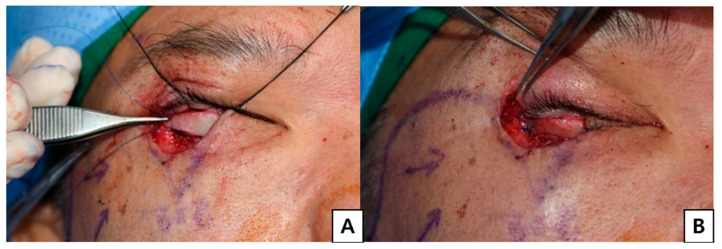
Posterior lamellar reconstruction using chondromucosal graft. (**A**) The nasal septal chondromucosal graft was sufficiently harvested and trimmed to allow tension-free coverage of the posterior lamellar defect before inset. (**B**) After insetting of the chondromucosal graft.

After assessing the defect, a nasal septal chondromucosal graft slightly larger than the tarsal plate defect was harvested to permit minor trimming and secure fixation (Figure 1). The graft consisted of the septal cartilage with the mucosa on a single surface, and the contralateral septal mucosa was left in situ to preserve the intranasal lining. The graft was inset with the mucosal surface oriented to replace the conjunctiva and cartilaginous components, substituting for the missing tarsus and thereby reconstructing the posterior lamella (Figure 2).

The nasal mucosa was sutured to the conjunctiva and the septal cartilage was sutured to the remaining tarsal plate using Vicryl# 6-0 buried sutures placed without tension (Video S1)

**Figure 3:**
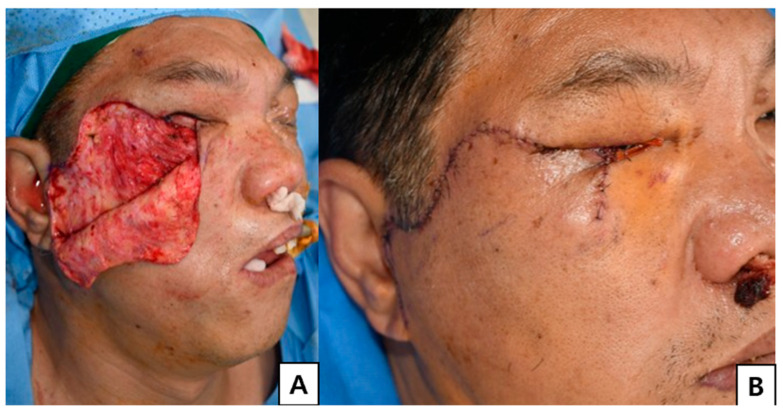
Anterior lamellar reconstruction with cheek rotation flap. (**A**) For anterior lamellar reconstruction, a Mustardé cheek rotation flap was designed as large as possible to maximize tissue availability and ensure robust vascularity. (**B**) Immediate postoperative view.

Reconstruction of the anterior lamella was tailored to the size of the defect using either a Mustardé cheek rotation flap for the lower eyelid or a Fricke flap for the upper eyelid, as appropriate. Local flap selection was based on defect location and size, and the flap was designed to be sufficiently large to ensure adequate vascularity and tension-free closure (Figure 3).

All key eyelid structures were reconstructed using these methods and temporary tarsorrhaphy was performed for ocular protection.

### 2.4. Donor-Site Management

Donor site management included buccal mucosal grafting, split-thickness skin graft (STSG), full-thickness skin graft (FTSG), and secondary healing using nasal packing.

**Figure 4:**
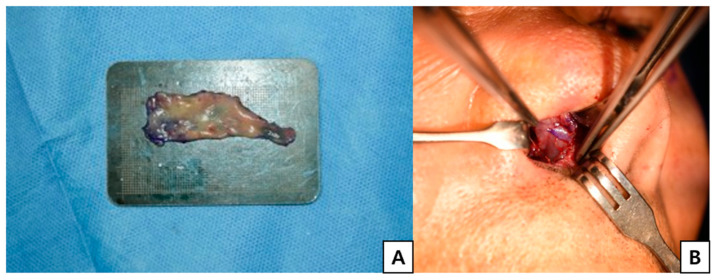
Donor site coverage using buccal mucosal graft. (**A**) Buccal mucosa was harvested to cover the donor site of the nasal septal chondromucosal graft. (**B**) Donor site coverage using the harvested buccal mucosal graft.

In patients who received a buccal mucosal graft, the graft was harvested from the buccinator region, with the donor site primarily closed. The edges of the harvested mucosa were anchored to the septal defect using 5-0 Vicryl sutures (Figure 4). A silastic sheet was then applied bilaterally to the nasal septum, followed by placement of an air Merocel^®^ packing (Medtronic, Jacksonville, FL, USA).

In the patients who underwent STSG or FTSG, the grafts were secured to the septal defect using 5-0 Vicryl anchoring sutures, followed by silastic sheet application and bilateral air Merocel packing.

In the single case managed by secondary healing, only air Merocel^®^ packing was placed without graft coverage.

### 2.5. Postoperative Care

All seven patients were maintained with a light compressive dressing, which was changed on postoperative days (POD) 1, 3, 5, and 7. During each dressing change, the color of the graft mucosa was carefully examined to assess graft viability. Tarsorrhaphy was removed on postoperative day 4, and complete suture removal was performed on POD 7; however, the Vicryl sutures placed between the mucosa and conjunctiva were left in place. Nasal packing was removed on POD 4 in patients who underwent graft coverage, and on POD 7 in patients managed by secondary healing. Silastic sheets were removed from both nasal cavities on POD 7. The buried 5-0 Vicryl sutures used for graft anchoring were left in place. On POD 10, the composite graft placed in the eyelid was evaluated to confirm graft uptake, and the grafted nasal septum donor site was examined to assess the healing status. Postoperative follow-up evaluations were performed at 1, 3, and 6 months to assess eyelid closure, ocular surface protection, and postoperative complications, including entropion, ectropion, and contracture.

### 2.6. Outcome Measures

Functional outcomes were assessed based on the following:

(1) ability to achieve complete eyelid closure
(2) preservation of ocular surface protection
(3) absence of complications such as ectropion, entropion, or corneal irritation

Graft outcomes were evaluated based on survival of the chondromucosal graft.

Donor-site morbidity was assessed based on healing status, crusting, dryness, airway discomfort, and septal perforation.

All patients were followed for at least 12 months.

## 3. Results

### 3.1. Patient Characteristics

Seven patients (five women and two men; mean age, 74 years; range, 57–89 years) underwent eyelid reconstruction using a combination of a nasal septal chondromucosal graft and either a Mustardé cheek rotation flap or a Fricke flap coverage designed to be as large as possible.

The mean follow-up period was approximately 3 years (range, 1–7 years), and all patients completed at least 12 months of follow-up. None of the patients experienced malignancy recurrence or flap/graft failure during the observation period (Table 1).

### 3.2. Defect Characteristics

Six patients developed eyelid defects following tumor resection (sebaceous carcinoma, squamous cell carcinoma, or basal cell carcinoma), and one patient presented with a post-burn eyelid defect.

The defect size was at least half of the horizontal eyelid length in all patients, and one case involved more than two-thirds of the eyelid. The defect location was the upper eyelid in three cases, the lower eyelid in three cases, and both the upper and lower eyelids in one case (Table 1).

### 3.3. Surgical Procedures Performed

Each reconstruction was planned according to the characteristics of the eyelid defect. Three patients underwent a Mustardé cheek rotation flap with buccal mucosal graft coverage of the donor site, two patients received a Fricke forehead flap combined with either a buccal mucosal graft or a split-thickness skin graft, one patient underwent a Mustardé flap with a full-thickness skin graft, and one patient underwent a Mustardé flap with secondary healing of the donor site.

### 3.4. Graft and Flap Outcomes

The chondromucosal graft sites were evaluated on postoperative days (POD) 1, 3, 5, 7, and 10. Until POD 3, all grafts appeared pale. In four patients, early ingrowth of fine vessels was observed on POD 5, and by POD 10, the grafts demonstrated a pinkish appearance consistent with graft take. In the remaining three patients, although the grafts were no longer pale on POD 5, clear neovascularization was not evident until POD 7; however, by POD 10, these grafts also showed a pinkish appearance, indicating successful graft take.

All patients (7/7) achieved complete graft survival without partial or total graft loss.

**Figure 5:**
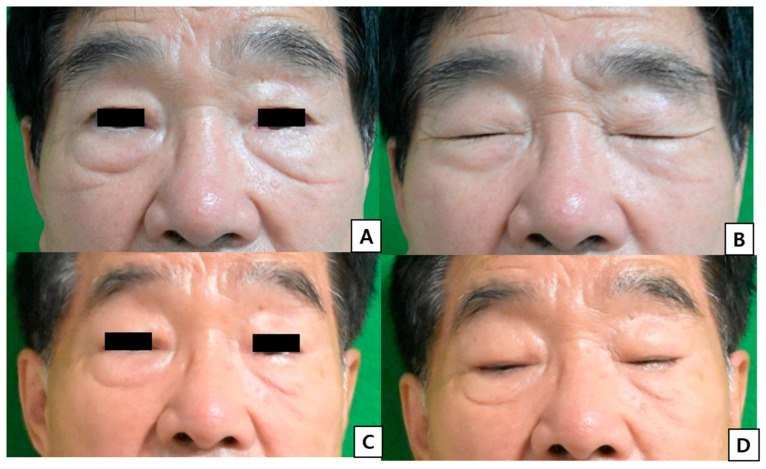
Postoperative outcomes after one-stage eyelid reconstruction. (**A**) Postoperative view at 3 months with eyes open. (**B**) Postoperative view at 3 months with eyes closed. (**C**) Postoperative view at 6 months with eyes open. (**D**) Postoperative view at 6 months with eyes closed.

All patients (7/7) achieved complete eyelid closure without lagophthalmos. No cases of ectropion, entropion, or persistent ocular irritation were observed. Eyelid symmetry was clinically acceptable in all patients at the final follow-up (Figure 5).

### 3.5. Complications

Early postoperative complications such as hematoma, infection, wound dehiscence, corneal ulcers due to corneal irritation, and partial graft loss were not observed.

No major or minor complications, including hematoma, infection, wound dehiscence, corneal ulceration, or graft loss, were observed.

### 3.6. Donor-Site Outcomes

**Figure 6:**
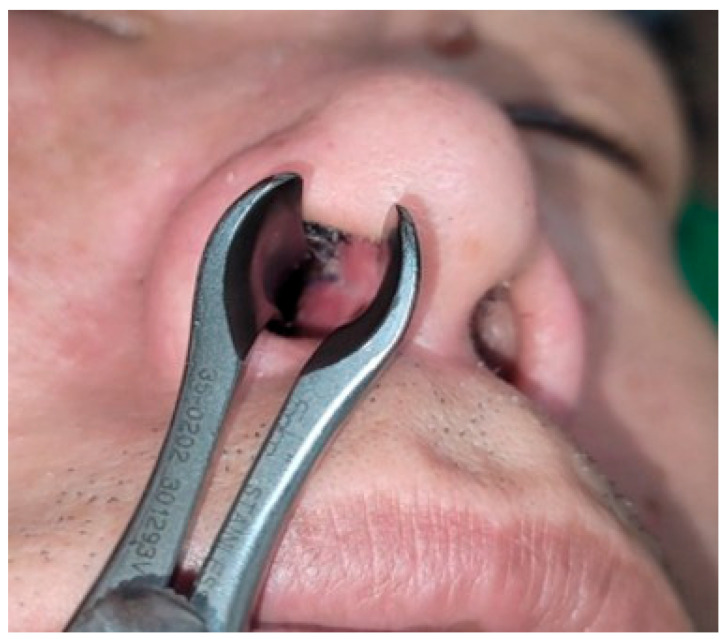
Complete healing of chondromucosal graft donor site.

Donor-site healing was generally uneventful. In the four patients who received a buccal mucosal graft, graft take was nearly complete by POD 7, with smooth healing and no evidence of septal perforation, infection, or airway problems related to crust formation or contracture. The oral mucosal donor sites healed uneventfully, with no discomfort or other morbidity at the 1-, 3-, and 6-month follow-up visits (Figure 6).

In contrast, the patient who underwent a split-thickness skin graft achieved graft take on POD 7 but developed mild contraction and noticeable crusting at approximately 1 month, which gradually resolved by 6 months. The patient who underwent a full-thickness skin graft achieved complete graft take on POD 10. Although contraction was less pronounced than that observed with split-thickness skin grafting, mild crusting and dryness-related discomfort were noted at approximately 1 month and improved by 6 months. No infections or septal perforations were observed in these cases.

In the patient managed with secondary healing, partial septal perforation developed, and complete healing required more than four weeks.

### 3.7. Secondary Procedures

The only secondary procedure was required in one patient with burns who developed postoperative scar contracture and subsequently underwent two additional scar release operations. None of the other patients required revision surgery.

## 4. Discussion

Owing to the unique anatomical and functional complexities of the eyelid, reconstruction remains one of the most demanding challenges in plastic and reconstructive surgeries. The eyelid comprises the anterior and posterior lamellae, which together provide ocular protection, lubrication, and aesthetics.

Eyelid defects have diverse etiologies, including trauma, tumor excision, burns, and congenital anomalies, and may vary from partial-thickness loss to extensive full-thickness defects [3]. While small partial-thickness defects can often be managed by primary closure or local advancement, larger full-thickness defects involving one-third to one-half of the horizontal lid require more complex techniques to reconstruct both the anterior and posterior lamellae [4]. However, because of the intricate anatomy and limited local tissue availability, the outcomes of such extensive reconstructions are frequently suboptimal, with risks of malposition, poor eyelid closure, corneal irritation, and other complications [3].

Various techniques have been described for posterior lamellar reconstruction, including the use of tarsoconjunctival grafts, auricular cartilage, hard palate mucosa, and nasal septal chondromucosal grafts [10]. Similarly, various flaps such as the Mustardé cheek rotation flap, Fricke flap, and Cutler–Beard flap have been employed for anterior lamellar repair [11].

Compared with previously reported techniques such as tarsoconjunctival flaps and hard palate grafts, our approach enables simultaneous reconstruction of both lamellae in a single stage while providing adequate structural support and a mucosal surface suitable for ocular contact.

However, reports combining posterior lamellar grafting with robust anterior flap coverage in a single-stage procedure are limited.

One challenge is the use of composite grafts in posterior lamellar reconstruction. Unlike skin grafts, composite grafts are thicker and rely entirely on imbibition and neovascularization from the recipient bed. Consequently, they are prone to partial necrosis or failure, especially in the presence of tension or inadequate vascularity [12]. Previous studies have reported failure rates exceeding 50% for grafts larger than 1.5 cm [8]. Therefore, the composite graft take is inherently less predictable, especially for larger defects. This limitation highlights the importance of adequate vascular support for successful graft survival.

In contrast, in the present study, all patients (7/7) achieved complete graft survival without partial or total graft loss, suggesting that adequate vascularized flap coverage may enhance graft viability even in relatively large defects.

Moreover, given the unique anatomical characteristics of the eyelid conjunctiva, the type of graft employed can directly influence ocular surface morbidity. Certain grafts, particularly those with irregular or keratinized surfaces such as the hard palate mucosa, have been associated with corneal irritation, epithelial defects, and even keratopathy. Therefore, careful selection of a posterior lamellar substitute is essential to minimize ocular surface complications and preserve corneal health [13,14].

In our series, no cases of corneal complications or persistent ocular irritation were observed, which may be attributed to the non-keratinized mucosal surface of the nasal septal chondromucosal graft.

We aimed to overcome these limitations by designing a novel procedure: a tension-free inset of a nasal septal chondromucosal composite graft for posterior lamellar replacement, combined with robust vascularized coverage using a large local flap for the anterior lamella. By maximizing the flap size and ensuring adequate vascularity, this method provides a favorable environment for composite graft survival, even in cases involving relatively large defects. This approach allows for tension-free reconstruction with sufficient vascular support, which may contribute to stable graft integration and favorable functional outcomes.

Furthermore, the nasal septal chondromucosal graft offers a distinct advantage because its non-keratinized mucosal surface closely resembles that of the native conjunctiva. Owing to these histological similarities, it is well tolerated when in direct contact with the cornea, thereby minimizing the risk of corneal irritation that may occur with keratinized grafts, such as the hard palate mucosa.

In addition, we addressed donor-site morbidity, a recognized limitation of harvesting nasal septal chondromucosal grafts that may result in septal perforation, hematoma, infection, airway problems caused by crust formation, or contracture, by introducing buccal mucosal grafting for septal defect management. Donor-site outcomes in our series were generally favorable, particularly in patients who underwent buccal mucosal grafting, with no septal perforation or significant airway-related complications observed.

This study has several limitations. First, the sample size was small due to the rarity of full-thickness eyelid defects requiring this specific reconstructive approach. Second, the retrospective design may introduce selection bias. Third, the absence of a control group limits direct comparison with alternative techniques. Finally, standardized outcome measures were not used, which may limit the objectivity of outcome assessment.

## 5. Conclusions

This study demonstrates that the integration of a nasal septal chondromucosal graft with well-vascularized local flap coverage, combined with buccal mucosal repair of the donor site, is a feasible and reproducible single-stage strategy for full-thickness eyelid reconstruction. The approach provided stable structural support, reliable graft survival, and satisfactory functional outcomes, while maintaining favorable donor-site healing. Although limited by a small sample size and retrospective design, this technique may represent a useful option for selected patients.

## Figures and Tables

**Table 1:** Patient demographics and clinical characteristics.

Patient	Age (Year)	Sex	Underlying Disease	Ocular or Nasal Surgery History	Etiology of Defect	Defect Site	Defect Size	Reconstructive Method			Follow Up (Year)
								Posterior	Anterior	Donor Site	
1	87	F	HTN	None	Tumor resection (sebaceous carcinoma)	Right upper lateral eyelid	1/2 of eyelid	NCMG	Fricke flap	Buccal mucosal graft	3
2	57	M	HTN, DM	Nasal polyp removal, septoplasty	Tumor resection (SCC)	Right lower lateral eyelid	>1/2 of eyelid	NCMG	Mustardé flap	Buccal mucosal graft	3
3	72	M	None	None	Tumor resection (BCC)	Left lower mid eyelid	1/2 of eyelid	NCMG	Mustardé flap	Buccal mucosal graft	2
4	89	F	HTN, asthma, pulmonary disease	None	Tumor resection (SCC)	Right upper mid eyelid	>1/2 of eyelid	NCMG	Fricke flap	Buccal mucosal graft	2
5	64	F	HTN, DM	None	Trauma (burn)	Right upper mid eyelid	>2/3 of eyelid	NCMG	Fricke flap	STSG	7
6	67	F	HTN	None	Tumor resection (BCC)	Right upper and lower lateral eyelid	1/2 of eyelid (both)	NCMG	Mustardé flap	Secondary healing	1
7	81	F	HTN, DM	None	Tumor resection (BCC)	Right lower lateral eyelid	1/2 of eyelid	NCMG	Mustardé flap	FTSG	7

Abbreviations: HTN, hypertension; DM, diabetes mellitus; NCMG, nasal septal chondromucosal graft; STSG, split-thickness skin graft; FTSG, full-thickness skin graft; SCC, squamous cell carcinoma; BCC, basal cell carcinoma.

## Data Availability

The data presented in this study are available from the corresponding author upon reasonable request.

## Supplementary Materials

The following supporting information can be downloaded at https://www.mdpi.com/article/10.3390/jcm15093190/s1. Video S1: Insetting of nasal septal chondromucosal graft.
